# Personalizing humanized mice with cells obtained from an HIV post treatment controller

**DOI:** 10.1186/1471-2334-14-S2-P36

**Published:** 2014-05-23

**Authors:** Ilona Hauber, Julian Schulze zur Wiesch, Udo Schuhmacher, Carola Schäfer, Jan van Lunzen, Joachim Hauber

**Affiliations:** 1 Heinrich Pette Institute, Leibniz Institute for Experimental Virology, Eppendorf, Germany; 2 University Medical Center Hamburg-Eppendorf, 20251 Hamburg, Germany

## Introduction

HIV research greatly benefits from the use of immunodeficient mouse systems that are susceptible to HIV target cells. Usually, immunodeficient mice are transplanted with primary human cells to develop a “human” immune system. Following engraftment, the animals are subsequently infected with HIV. Here, we applied a different approach and personalized mice by directly transplanting cells from an HIV-infected patient.

## Materials and methods

CD4+ T cells were isolated from a HIV-infected post treatment controller (PTC) and transplanted into 6 week old Rag2-/-γc-/- (Rag-hu) mice in order to monitor virus replication over time.

Upon presentation to the clinic in 1998, the patient’s viral load was 52.000 HIV copies/ml with near-normal CD4+ T cell count of 491/ul and slightly reduced CD4+/CD8+ ratio of 0,4. Treatment with AZT, 3TC and effavirenz was initiated and the viral load was undetectable after few weeks and undetectable for the next couple of years. In May 2004 therapy was stopped after more then 5 years in an attempt of STI (structured treatment interruption). Remarkably, the viral load stayed below the level of detection with stable CD4+ counts since then. The patient was further analyzed for host factors but neither the HLA molecules (HLA A 01,02, B44, B52) nor a deletion of the CCR5 receptor could account for the control of the virus after treatment interruption.

## Results

At 8 weeks after transplantation the mice were engrafted with 15-30% human CD45+CD3+ and significant viral loads (from 3 - 62 x 10E6) were detected. Analysis of lymphoid cells of the spleen, bone marrow, liver and salivary gland demonstrated a fully established HIV infection.

## Conclusion

The direct transplantation of patient-derived cells into hemato-lymphoid system mice provides a novel experimental approach to analyze the presence of replication-competent HIV in patients with undetectable viral loads such as, for example, post treatment controllers (PTC).

